# Step by step male to female transsexual surgery

**DOI:** 10.1590/S1677-5538.IBJU.2017.0044

**Published:** 2018

**Authors:** Rodrigo Uliano Moser da Silva, Fernando Jahn da Silva Abreu, Gabriel M. V. Da Silva, João Vitor Quadra Vieira dos Santos, Nelson Sivonei da Silva Batezini, Brasil Silva, Tiago Elias Rosito

**Affiliations:** 1 Departamento de Urologia, Hospital de Clínicas de Porto Alegre, Porto Alegre, RS, Brasil

## Abstract

**Introduction:**

After the diagnosis of transsexualism is confirmed therapy commences with psychotherapeutic preparation for the conversion, and after conversion, long-term patient rehabilitation is maintained for at least two years. The indication for surgery is chronic discomfort caused by discord with the patient's natural gender, intense dislike of developing secondary sex characteristics and the onset of puberty. The surgical conversion of transsexuals is the main step in the complex care of these problematic patients (1). This surgery was first described by Benjamin H, using a flap of inverted penile skin (2) and is considered the gold standard since then. Male-to-female transsexual surgical techniques are well defined and give good cosmetic and functional results. Sex reassignment surgery promotes the improvement of psychological aspects and social relationships as shown in the World Health Organization Quality of Life Assessment applied in the patients submitted to this procedure (3). Techniques include the creation of a normal appearing female introitus, a vaginoplasty allowing sexual intercourse and the capability of clitoral orgasm (4). Various methods for neovaginoplasty have been described and can be classified into five categories, i.e. pedicled intestinal transplants, penile skin grafts, penile skin flaps, non-genital skin flaps and non-genital skin grafts (5). In our Hospital, we use penile and scrotal skin flaps. Until now, 174 procedures have been performed by our team using this technique with high rates of satisfaction (3).

**Patients and methods:**

We present a step-by-step male to female transsexual surgery.

**Conclusion:**

Surgical gender reassignment of male transsexuals resulted in replicas of female genitalia which enabled coitus with orgasm (1). With this video we show step by step that a surgery using penile skin flaps is able to be performed with good cosmetic results.

## ARTICLE INFO

**Available at: http://www.intbrazjurol.com.br/video-section/20170044_Silva_et_al/**

**Int Braz J Urol. 2018; 44 (Video #6): 407-8**
